# Biofabrication of Fe_3_O_4_ Nanoparticles from *Spirogyra hyalina* and *Ajuga bracteosa* and Their Antibacterial Applications

**DOI:** 10.3390/molecules28083403

**Published:** 2023-04-12

**Authors:** Muhammad Shakeeb Sharif, Hajra Hameed, Abdul Waheed, Muhammad Tariq, Afshan Afreen, Asif Kamal, Eman A. Mahmoud, Hosam O. Elansary, Saddam Saqib, Wajid Zaman

**Affiliations:** 1 Department of Biotechnology, Mirpur University of Science and Technology, Mirpur 10250, Pakistan; mshakeebsharif@gmail.com (M.S.S.); hajrahameedmughal@gmail.com (H.H.);; 2 Agricultural Genomics Institute at Shenzhen, Chinese Academy of Agricultural Sciences, Shenzhen 518120, China; 3 Department of Plant Sciences, Quaid-i-Azam University, Islamabad 45320, Pakistan; 4 Department of Food Industries, Faculty of Agriculture, Damietta University, Damietta 34511, Egypt; 5 Department of Plant Production, College of Food & Agriculture Sciences, King Saud University, P.O. Box 2460, Riyadh 11451, Saudi Arabia; 6 State Key Laboratory of Systematic and Evolutionary Botany, Institute of Botany, Chinese Academy of Sciences, Beijing 100093, China; 7 University of Chinese Academy of Sciences, Beijing 100049, China; 8 Department of Life Sciences, Yeungnam University, Gyeongsan 38541, Republic of Korea

**Keywords:** *Ajuga bracteosa*, *Spirogyra hyalina*, nanoparticles, antibacterial, antioxidant

## Abstract

Iron oxide nanoparticles (NPs) have attracted substantial interest due to their superparamagnetic features, biocompatibility, and nontoxicity. The latest progress in the biological production of Fe_3_O_4_ NPs by green methods has improved their quality and biological applications significantly. In this study, the fabrication of iron oxide NPs from *Spirogyra hyalina* and *Ajuga bracteosa* was conducted via an easy, environmentally friendly, and cost-effective process. The fabricated Fe_3_O_4_ NPs were characterized using various analytical methods to study their unique properties. UV-Vis absorption peaks were observed in algal and plant-based Fe_3_O_4_ NPs at 289 nm and 306 nm, respectively. Fourier transform infrared (FTIR) spectroscopy analyzed diverse bioactive phytochemicals present in algal and plant extracts that functioned as stabilizing and capping agents in the fabrication of algal and plant-based Fe_3_O_4_ NPs. X-ray diffraction of NPs revealed the crystalline nature of both biofabricated Fe_3_O_4_ NPs and their small size. Scanning electron microscopy (SEM) revealed that algae and plant-based Fe_3_O_4_ NPs are spherical and rod-shaped, averaging 52 nm and 75 nm in size. Energy dispersive X-ray spectroscopy showed that the green-synthesized Fe_3_O_4_ NPs require a high mass percentage of iron and oxygen to ensure their synthesis. The fabricated plant-based Fe_3_O_4_ NPs exhibited stronger antioxidant properties than algal-based Fe_3_O_4_ NPs. The algal-based NPs showed efficient antibacterial potential against *E. coli*, while the plant-based Fe_3_O_4_ NPs displayed a higher zone of inhibition against *S. aureus*. Moreover, plant-based Fe_3_O_4_ NPs exhibited superior scavenging and antibacterial potential compared to the algal-based Fe_3_O_4_ NPs. This might be due to the greater number of phytochemicals in plants that surround the NPs during their green fabrication. Hence, the capping of bioactive agents over iron oxide NPs improves antibacterial applications.

## 1. Introduction

Metallic oxide nanoparticles have revolutionized the field of nanotechnology by furnishing fundamental biomedical applications to resolve various complex problems [1,2,3]. These nanoparticles have developed into promising biomedicinal agents for diverse purposes, including targeted drug delivery [4], tissue engineering, bio-sensing [5], imaging [6], and wound healing [7,8]. The green fabrication of iron oxide nanoparticles (Fe_3_O_4_ NPs) has achieved outstanding significance in the biomedical sector in a sustainable manner [9,10]. These nanoparticles are superparamagnetic, biodegradable, sustainable, and have an elevated surface area [11,12]. Iron oxide nanoparticles (NPs) are generally utilized in the pharmaceutical industry to produce various efficacious drugs used for the prevention of infections [13]. Iron oxide nanoparticles are used as potent therapeutic and theranostic agents and have superb biomedical engineering applications due to their magnetic properties [13,14]. After the surface modification of iron oxide NPs, they have been applied widely for diagnosis, envisaging melanoma cells, visualizing severe metastases in the liver, and as a blood pool agent for angiographic purposes [15]. Fe_3_O_4_ NPs have been used to advance novel, extensive-spectrum antimicrobials against various pathogenic microbes [16]. Furthermore, Fe_3_O_4_ NPs have shown strong antioxidant [17], antifungal, antiviral, antibacterial, and anti-inflammatory activities [18].

The fabrication of NPs via green synthesis has attracted considerable attention because it is the easiest, most sustainable, reliable, and eco-friendly method [19]. Green synthesis is a potential alternative to the chemical process where non-ecofriendly products are released with adverse and potentially lethal effects [20,21,22]. The fabrication of NPs via chemical methods requires high temperatures or pressures to initiate the chemical reaction and gain the necessary stability for potent toxic stabilizers [23,24,25]. In contrast to chemical synthesis, the green strategy proved inexpensive and nontoxic. Moreover, green-synthesized NPs are promising in the biomedical arena because they are less detrimental to human health than chemically synthesized NPs [26]. The potency of green biofabricated NPs has been attributed to various phytochemicals used to reduce the metal salt, cap the NPs during their synthesis, and contribute efficaciously to the fabrication of potent NPs [27]. NPs created via green synthesis have achieved significant value for researchers because of their unique features [28]. Therefore, protocols to produce harmless metallic oxide NPs are essential.

The green fabrication of metallic oxide NPs using various bio-reductants involving microorganisms, fungi, and algal and plant extracts has been previously reported by diverse researchers [29]. Algal and plant sources have been significantly explored for the fabrication of metallic oxide NPs and are frequent options for many researchers because of their convenience and accessibility. Furthermore, algae and plants are stockpiles of chief phytochemicals, such as carotenoids, flavonoids, and phenolic compounds, which may confer various biological features to the NPs [30]. Many researchers have reported the fabrication of Fe_3_O_4_ NPs from algal and plant extracts, such as *Petalonia fascia, Colpomenia sinuosa, Padina pavonica, Moringa oleifera, Ananas comosus, Perilla frutescens, Mimosa pudica, Acacia mearnsii*, and *Caricaya papaya*. The green fabrication of Fe_3_O_4_ NPs was safe, sustainable, and eco-friendly [31].

The *Spirogyra hyalina* is a green alga belonging to the family Zygnemataceae and it is abundant in freshwater regions throughout the world [32]. There is strong evidence that *S. hyalina* is commercially and economically prominent as it is employed in the production of bioethanol in a fermentative process with *Zymomonas mobilis* [33]. This alga played a vital role in decreasing the degree of salinity in seawater toward freshwater by biosorption [34]. It also has the potential to overcome the diverse organic waste and monitor water quality more effectively in an optimized way. Dried biomass has an aptitude for eliminating heavy metals from bodies of water, such as Cd, Pb, Hg, and Co, by biosorption [35]. *Spirogyra* sp. is a rich source of antioxidants because of various phytochemicals, such as terpenoids, saponins, tannins, and flavonoids [22]. *Ajuga bracteosa* is a perennial evergreen herbaceous plant located in hilly regions and belongs to the family Lamiaceae. Because it contains essential pharmacologically active agents, it is an important medicinal plant in the Himalayan region for a variety of ailments. The species is grown at a 2000 m altitude in subtropical and temperate regions around the world, and it is found in Pakistan’s northern sectors [36]. The respective plant is used to treat various skin ailments, respiratory disorders, alimentary issues, and malarial problems. *A. bracteosa* is highly prevalent, particularly as a purifier of blood, a diaphoretic, cough relief, an appeaser of asthma, and a palliator of asthma. The bark is utilized to overcome sore throat and jaundice [37]. It also has a good scavenging effect, antimicrobial potency, and anticancer and antimalarial activities [38]. Previously, the antioxidant potential of *A. bracteosa* was attributed to various biological phytochemicals, including flavonols, neo-clerodane, glycosides, diterpenoids, ergosterols, phytoecdysones, and iridoid glycosides [39].

This paper reports the biofabrication of Fe_3_O_4_ NPs by exploiting the biomolecules of *Spirogyra hyalina* and *Ajuga bracteosa* as bioreductants. Moreover, the capping of diverse phytochemicals on Fe_3_O_4_ NPs may enhance the bioactivities of these biofabricated NPs. The green-fabricated Fe_3_O_4_ NPs were characterized using different analytical methods to determine their unique properties. Furthermore, the present study also explored the biomedicinal potential of green-synthesized Fe_3_O_4_ NPs and algal and plant extracts by accessing their antibacterial and antioxidant activities. The outcomes revealed the enhanced biological potential of Fe_3_O_4_ NPs, which might be because the capping of bioactive agents around NPs is present. Nevertheless, further research will be needed to understand the chemistry of metallic oxide NPs.

## 2. Results

### 2.1. Characterization of Iron Oxide NPs

The Fe_3_O_4_ NPs were biofabricated using the algal and plant extracts of *S. hyalina* and *A. bracteosa*. In the case of algal-based Fe_3_O_4_ NPs, the color change from yellow to reddish-brown was observed. The instantaneous dark brownish color change from light yellow confirmed the fabrication of plant-based Fe_3_O_4_ NPs. The color change was attributed to surface plasmon resonance and the interaction of biomolecules with metal ions. These biosynthesized NPs were analyzed via various analytical techniques.

#### 2.1.1. UV-Vis Analysis of Fe_3_O_4_ NPs

**Figure 1:**
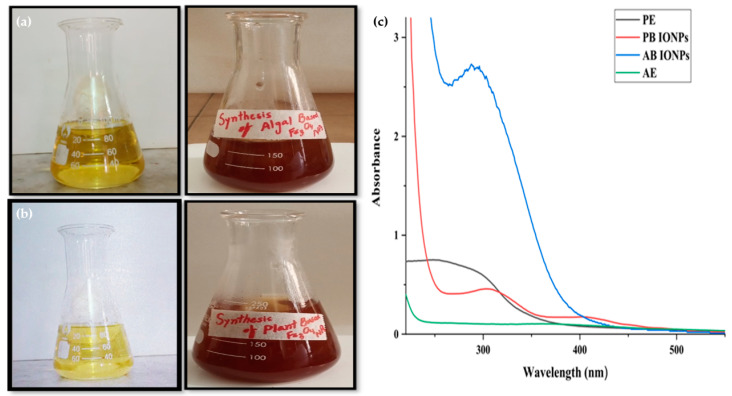
(**a**). A color change from the dark yellow of the algal extract to reddish-brown is observed during the biofabrication of algal-based IONPs. (**b**). The light yellow color of the plant extract changed into a dark brown during the biosynthesis of plant-based IONPs. (**c**). UV-Vis spectrum of algae extract, plant extract, and algal and plant-based Fe_3_O_4_ NPs.

UV-visible spectroscopy was used to monitor and approve the fabrication of Fe_3_O_4_ NPs. The absorption peaks of biofabricated Fe_3_O_4_ NPs were observed because surface plasmon vibrations reached the excitation stage. The figure shows that algae and plant extracts had no distinct peaks. In the case of algal-based Fe_3_O_4_ NPs, the sharp peak was displayed at 289 nm. While in the case of plant-based Fe_3_O_4_ NPs, the peak value for absorbance was observed at 306 nm shown in Figure 1. Many studies revealed the maximal absorbance at 250, 291, 297, 300, 328, and 330 nm for Fe_3_O_4_ NPs synthesized from algae and plants, including *Petalonia fascia* [40], *Ficus carica* [41], *Mimosa pudica* [42], *Moringa oleifera* [31], *Bauhinia tomentosa* [43], and *Phyllanthus niruri* [44], which supported these findings. These variations of maximal absorbance were attributed to the capping of biomolecules on NPs. These biomolecules affect the size of NPs through bioreduction, which also affects maximal absorbance [30,31].

#### 2.1.2. FTIR Analysis of Fe_3_O_4_ NPs

**Figure 2:**
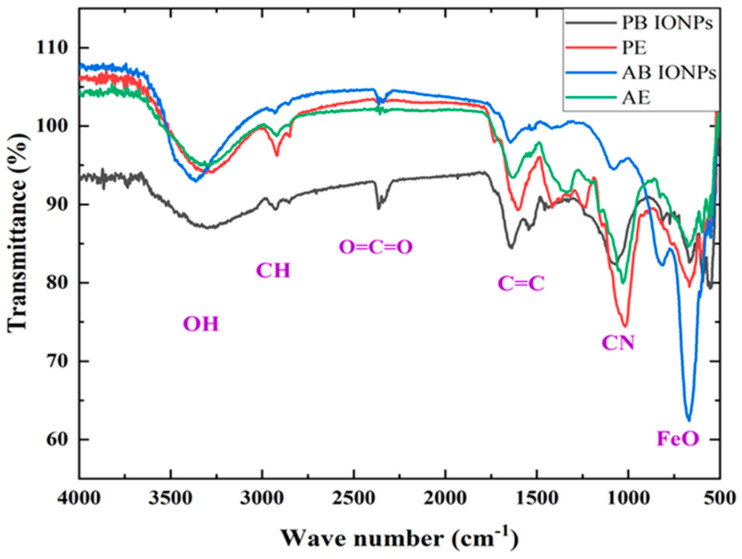
FTIR spectra of AE, PE, and biofabricated algal and plant based Fe_3_O_4_ NPs show various functional groups for biomolecules from *Ajuga bracteosa* and *Spirogyra hyalina* involved in the bioreduction of NPs.

FTIR spectroscopy was performed to determine the functional groups of various biomolecules interacting with the fabricated Fe_3_O_4_ NPs. The FTIR spectra revealed the active biomolecules present in the algal and plant extracts that had played an essential role in reducing the precursor salt during the fabrication of iron oxide NPs and maintaining their stability. In the FTIR spectrum of the algal extract, shown in Figure 2, the prominent peak detected at 3313 cm^−1^ corresponded to the stretch of the O–H group of either alcohol or algal phenolics. Kale et al. also reported the presence of an O–H bond close to this position [37]. The peaks at 2924 cm^−1^ and 2852 cm^−1^ indicate the stretching vibration of a C–H bond (alkane) [45]. The C=C (alkene) stretching vibration was observed at 1641 cm^−1^ as a stretched vibration form. The peak at 1033 cm^−1^ was attributed to the stretching vibration of the primary amine C–N [34]. The FTIR spectrum of algal-based iron oxide NPs revealed a peak at 3373 cm^−1^ representing the O–H stretching vibration of alcohol or algal phenolics [35]. The peaks at 2932 cm^−1^ and 2852 cm^−1^ refer to the C–H stretched vibrations of an alkane. The peak at 2355 cm^−1^ was attributed to the absorption of atmospheric CO_2_, and the peak at 1646 cm^−1^ was assigned to the C=C or alkene stretching vibration [33]. The dominant peak at 674 cm^−1^ was attributed to the Fe–O in iron oxide, confirming the fabrication of NPs. The current result strongly agrees with the study reported by Aisida et al. [46].

The FTIR spectrum of the plant extract revealed a peak at 3305 cm^−1^, confirming the presence of the O–H bond in a stretched state for alcohol or plant phenolics [37]. The absorption peaks at 2918 cm^−1^ and 2854 cm^−1^ denote the C–H bond of the alkane in the mode of stretched vibrations [45]. The peak at 1609 cm^−1^ might be due to the C=C bond of an alkene in the stretched state [33]. In contrast, the absorption peak at 1020 cm^−1^ is probably due to the C–N bond of the amine in a stretched form [34]. The FTIR spectrum of plant-based iron oxide NPs suggests that there are diverse phytochemicals that play a vital role in the fabrication of iron oxide NPs. The peak at 3312 cm^−1^ was attributed to the O–H functional group of alcohol or plant phenolics. These findings are correlated with the study reported by Qasim et al. [45]. The absorption peaks at 2926 cm^−1^ and 2852 cm^−1^ were assigned to the stretching vibration of the alkane C–H group [36]. The peak at 2362 cm^−1^ was attributed to the absorption of CO_2_ in the sample. At 1635 cm^−1^, the absorption band corresponds to the C=C bond of an alkene in its stretched state [33]. The peak at 622 cm^−1^ is the typical peak of the Fe–O of iron oxide, confirming the fabrication of iron oxide NPs, and it is consistent with the findings reported by Liu et al. [47]. *A. bracteosa* has been used to incorporate diverse bioactive agents, such as ergosterol, diterpenoids, glycosides, phytoecdysones, and flavonols that assist the present FTIR spectra [38]. Many studies disclosed the existence of essential bioactive molecules in Fe_3_O_4_ NPs and their binding to the NPs [39].

#### 2.1.3. X-ray Diffraction of Fe_3_O_4_ NPs

**Figure 3:**
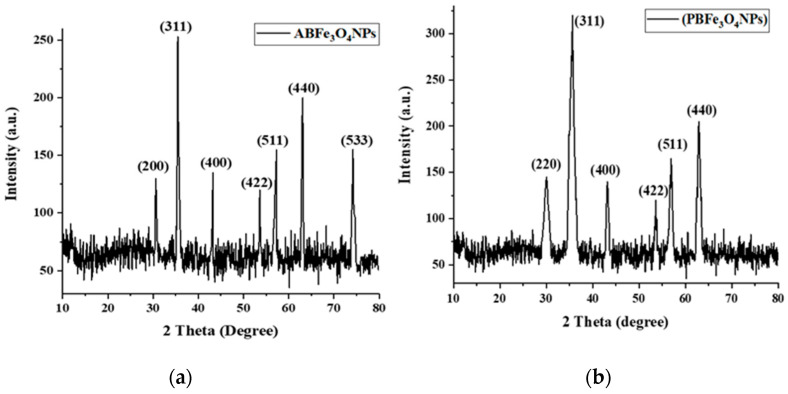
(**a**) XRD pattern of algal-based Fe_3_O_4_ NPs (**b**) XRD pattern of plant-based Fe_3_O_4_ NPs.

The XRD analysis of algal-based Fe_3_O_4_ NPs detected seven characteristic peaks for magnetite (Fe_3_O_4_) positioned at various angles of 2θ such as 30.55°, 35.45°, 43.25°, 53.61°, 57.27°, 63.1°, and 74.20°, corresponding to the (200), (311), (400), (422), (511), (440), and (533) planes, respectively, as shown in Figure 3a. The XRD peaks observed for the algal-based iron oxide NPs were similar to the standard magnetite XRD pattern in the ICCD file (00-003-0863), confirming the cubic structure of the crystalline system. The mean crystallite size of the respective NPs was 42 nm. The intense, sharp peaks showed that the plant-based Fe_3_O_4_ NPs were crystalline (Figure 3b). The XRD pattern revealed six peaks at 2θ of 30.06°, 35.63°, 43.13°, 53.62°, 56.90°, and 62.86°, which were assigned to the planes of (220), (311), (400), (422), (511), and (440), respectively. The XRD peaks were cross-referenced with the ICCD file, which has the number 00-019-0629. The average crystallite size of the plant-based iron oxide NPs, determined by the Debye–Scherrer formula, was 36 nm. Kale et al. (2018) biofabricated Fe_3_O_4_ NPs by exploiting the extract of *Cymbopogon citratus* via the reduction method. The XRD pattern showed six sharp peaks at various positions of 2θ, which corresponded to the (311), (220), (400), (422), (511), and (440) planes, which is consistent with the XRD findings of plant-based Fe_3_O_4_ NPs [37]. Yew et al., 2016 synthesized iron oxide NPs using the extract of *Kappaphycus alvarezii,* a seaweed. XRD revealed seven characteristic sharp peaks to confirm the crystalline nature of algal-based Fe_3_O_4_ NPs [38]. The broad XRD peaks might be linked to the small size of NPs [48].

#### 2.1.4. Scanning Electron Microscopy

**Figure 4:**
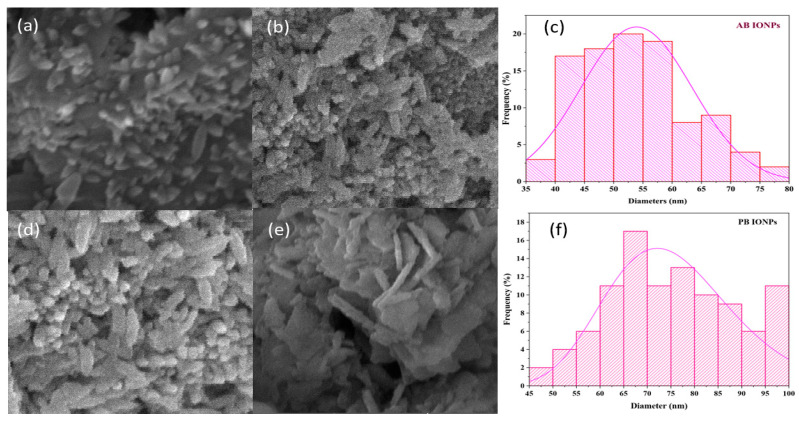
(**a**) SEM image of AB Fe_3_O_4_ low-resolution NPs (**b**). AB Fe_3_O_4_ NPs at high resolution (**c**). Size distribution histogram for AB Fe_3_O_4_ NPs (**d**) SEM image of PB Fe_3_O_4_ NPs at low resolution (**e**) PB Fe_3_O_4_ NPs at high resolution (**f**). Size distribution histogram for PB Fe_3_O_4_ NPs (Image J and Origin software were used to determine the particle size by selecting one hundred NPs).

Figure 4a,b show the morphology of fabricated algal-based Fe_3_O_4_ NPs. As shown in the SEM image, the synthesized NPs were spherical and spindle-shaped. The mean size of biosynthesized NPs was 52 nm (35–80 nm). A few clusters of agglomerates were also observed, probably due to the accretion of bioactive reductive agents in algal extract or the magnetic tendency of Fe_3_O_4_ NPs [40]. Figure 4d–e shows the morphology of plant-based NPs. The fabricated Fe_3_O_4_ NPs were spherical and rod-shaped. The mean size of the NPs was 75 nm (45–100 nm). Some of the NPs were in an aggregated form. The aggregation of NPs might be due to the accumulation of diverse reductive agents in plant extracts or the magnetic tendency of biofabricated Fe_3_O_4_ NPs. SEM confirmed the fabrication of spindle-shaped Fe_3_O_4_ NPs via forced hydrolysis with a size range of 60–250 nm [49]. Another study reported the fabrication of spherical-shaped Fe_3_O_4_ NPs from *Perilla frutescens*, approximately 50 nm in size, which is also consistent with the present findings [50]. Previous research reported that the size of the Fe_3_O_4_ NPs from the peel extract of plantains and the seed extract of pomegranates were 30–50 nm and 25–55 nm, respectively [51,52].

#### 2.1.5. EDX Analysis of Fe_3_O_4_ NPs

**Figure 5:**
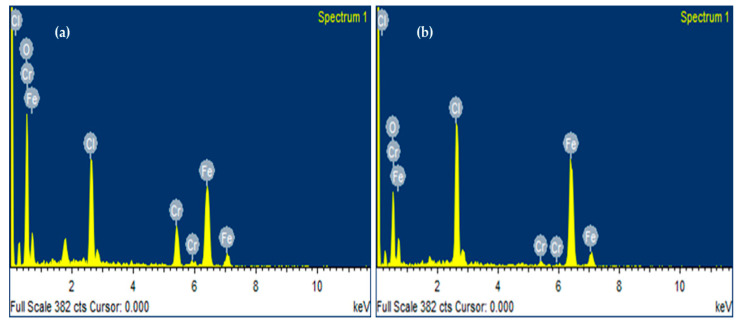
(**a,b**). EDX spectrum of biofabricated algal- and plant-based Fe_3_O_4_ NPs, showing sharp peaks for iron and oxygen. It also depicts other elements, such as chlorine and chromium, but with low mass percentages.

The EDX spectrum in Figure 5a revealed the intense peaks of iron at 6.4 keV and oxygen at 0.6 keV that affirmed the formation of Fe_3_O_4_ NPs. Their mass percentages were 36.27% and 38.47%, respectively. The chlorine peak was also observed in the EDS graph with a 14.33% mass. It was determined from Figure 5b that the EDX spectrum of plant-based Fe_3_O_4_ NPs were comprised of significant iron peaks at 6.4 keV and oxygen peaks at 0.6 keV, with mass percentages of 51% and 29%, respectively, confirming the fabrication of Fe_3_O_4_ NPs. Figure 5 shows other elements, such as Cl and Cr. The Cl peak was attributed to the ferric chloride salt precursor utilized to fabricate Fe_3_O_4_ NPs [21]. The chromium peak might be due to the sputtering of Fe_3_O_4_ NPs before SEM analysis. Sputter coating prevents specimen charging and increases the signal-to-noise ratio to produce better quality images [53]. The EDX analysis of the currently biofabricated Fe_3_O_4_ NPs is also consistent with other studies [54,55]. According to other studies, the expected values from EDX are closely related to the current findings [43].

### 2.2. In Vitro Studies

Different biological activity measures, such as the antibacterial and antioxidant properties of various fractions and Fe_3_O_4_ NPs, were used to demonstrate that these NPs performed better than algae and plant fractions.

#### 2.2.1. Antibacterial Activity

**Figure 6:**
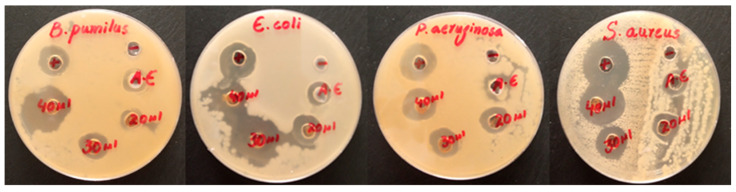
Antibacterial activity of algal-based Fe_3_O_4_ NPs against gram-positive and gram-negative bacteria.

**Figure 7:**
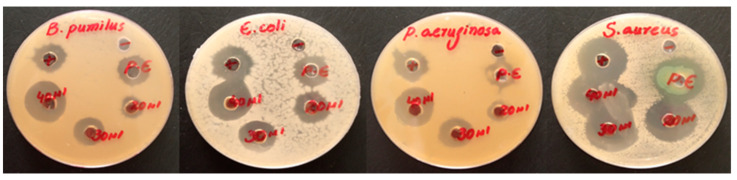
Antibacterial activity of plant-based Fe_3_O_4_ NPs against gram-positive and gram-negative bacteria.

**Figure 8:**
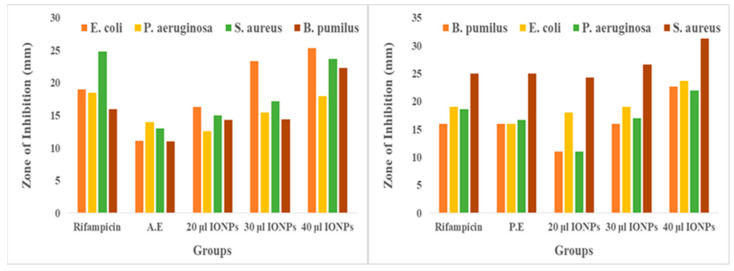
Antibacterial activity of positive controls, AE, PE, and algal- and plant-based Fe_3_O_4_ NPs, was evaluated by the well diffusion method against model gram-positive and gram-negative bacterial strains. Because the experiment was repeated three times, the values are reported as the mean ± SEM.

The antibacterial activity of the AE, PE, and Fe_3_O_4_ NPs was investigated for both gram-positive (*B. pumilus and S. aureus*) and gram-negative (*E. coli and P. aeruginosa*) bacteria. Based on the zone of inhibition produced, both algal and plant-based Fe_3_O_4_ NPs showed remarkable antibacterial activity compared to the algal and plant extracts against both types of investigated bacteria shown in Figure 6 and Figure 7. Overall, the antibacterial graphs shown in Figure 8 that plant-based Fe_3_O_4_ NPs had strong antibacterial potency compared to that of algal-based Fe_3_O_4_ NPs against the various types of gram-positive and gram-negative bacterial strains. This result might be due to the higher number of phytochemicals found in plant extracts compared to algal extracts.

In many studies, the same results of antibacterial potency were revealed by Fe_3_O_4_ NPs biofabricated from extracts of *Carcia papaya* [55], *Sida cordifolia* [56], *Purpureocillium platinum* [57], and *Phoenix dactylifera* [58] against gram-positive and gram-negative bacterial strains. These metallic oxide NPs may decrease the expression of an antibiotic-resistant gene in an antibiotic-resistant bacterium [59]. Iron oxide NPs have an aptitude for adsorption and penetration into bacterial biofilms because of their distinct physicochemical characteristics, such as hydrophobicity, surface charge, and large surface area [60]. Moreover, the respective NPs attach directly to the cell walls of various microorganisms and destroy themselves efficiently [58].

#### 2.2.2. Antioxidant Activity

**Figure 9:**
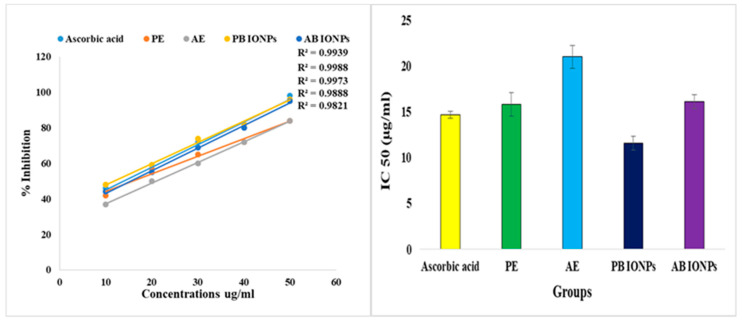
Antioxidant activity of AE, PE, and algal- and plant-based Fe_3_O_4_ NPs and the concentration needed to inhibit 50% of the scavenging activity of DPPH.

The scavenging activity of biofabricated Fe_3_O_4_ NPs was compared with algae and plant extracts determined by DPPH assay. The methanolic extract of *S. hyalina* and the aqueous extract of *A. bracteosa* showed IC_50_ values of 21 ± 1.23 µg/mL and 15.8 ± 1.3 µg/mL, *respectively.* Both algal- and plant-based Fe_3_O_4_ NPs showed remarkable scavenging potential (IC_50_ = 16.1 ± 0.74 µg/mL and 11.6 ± 0.76 µg/mL) compared to that of algal and plant extracts. A low IC_50_ value shows that the substance has a higher antioxidant potential. Plant-based Fe_3_O_4_ NPs outperformed the algal-based Fe_3_O_4_ NPs in terms of antioxidant potential. This might be due to the increased activity of phenolic molecules after the fabrication of Fe_3_O_4_ NPs shown in Figure 9. Phenolic active agents found in plant extracts play a crucial role in producing Fe_3_O_4_ NPs due to their high antioxidant activity and ability to act as reducing agents [47].

Plant-based Fe_3_O_4_ NPs exhibited better antioxidant potency than various oxidants that might be linked to plentiful bioactive agents [37]. The phytochemicals capped on the small-sized NPs may be amenable to the best scavenging activity by acting as a stabilizing agent [61]. The pharmacological features of bioactive agents accelerated the therapeutic efficacy of NPs with enhanced biological applications [53]. Another study investigated the scavenging effect of Fe_3_O_4_ NPs using the DPPH method and revealed the best antioxidant potential that supports these findings [17].

## 3. Materials and Methods

### 3.1. Preparation of Algal and Plant Extract

*Ajuga bracteosa* Wall ex Benth and *Spirogyra hyalina* were collected from Azad Kashmir, Pakistan, at coordinates 34.22° N 73.28° E in April 2022. A taxonomist of the Department of Biotechnology, Mirpur University of Science and Technology Mirpur, Azad Kashmir, Pakistan, identified the plant and algae. The voucher specimens (MUST1402- MUST1403) were deposited at the Herbarium of the Department of Biotechnology.

*Spirogyra hyalina* was collected from the freshwater region of Jatlan Head, district Mirpur, Azad Kashmir, Pakistan. The material was washed with distilled water to remove unwanted particles and placed in a dark area to maintain dryness. For the methanolic algal extract, the material was soaked in methanol for 72 h by keeping it in a shaker. After a specified period, the methanolic algae extract was obtained by concentrating it in a vacuum via a rotary evaporator. Subsequently, 10 g of algae was dissolved in 200 mL of distilled water and then filtered via Whatman filter paper No. 1 before being stored in a refrigerator for later use. The herbaceous plant, *Ajuga bracteosa*, was gathered from Samahni, District Bhimber, Azad Kashmir, Pakistan. The fresh plant parts were washed with distilled water to remove the undesirable particles. These were dried in the shade. The whole plant parts were minced into a fine powder, and 10 g of the minced powder was dissolved in 200 mL of distilled water. The suspension was placed on a hot plate at 60 °C for one hour with constant stirring. After the specified period, it was filtered through Whatman filter paper No. 1 before being stored in a refrigerator for later use. Previous studies also reported the green fabrication of iron oxide NPs using the methanolic and aqueous solutions of algal and plant extracts [56,57,58].

### 3.2. Green Biofabrication of Algal- and Plant-Based Iron Oxide NPs

**Figure 10:**
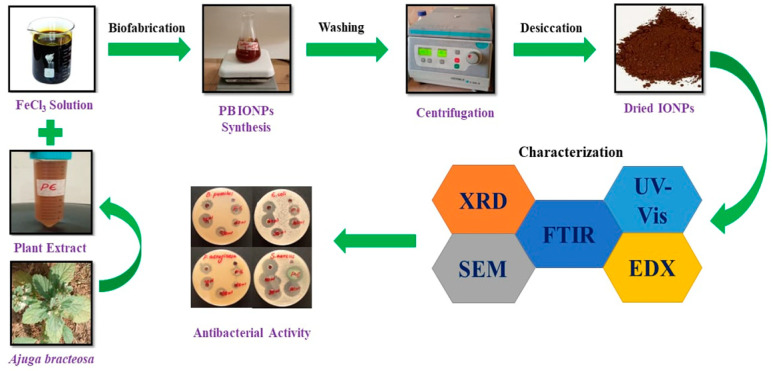
Green fabrication of iron oxide NPs using an aqueous extract of *Ajuga bracteosa*.

Ferric chloride hexahydrate was used as a salt precursor to fabricate IONPs. An amount of 1.35 g (0.1M) of FeCl_3_.6H_2_O was dissolved in 50 mL of distilled water in a bottom flask and heated for 20 min at 70 °C with mild stirring using a hot plate. Subsequently, 50 mL of the algal extract was added dropwise to the iron solution and heated to 60 °C for 30 min. The reddish-brown color that appeared indicates the formation of iron oxide NPs. The resulting product was centrifuged to remove impurities by washing two times with distilled water and three times with ethanol at 6000 rpm for 20 min. The final product was dried and stored for future use. In the case of the fabrication of iron oxide NPs from *Ajuga bracteosa*, the same salt precursor was applied shown in Figure 10. A 0.1 M FeCl_3_.6H_2_O solution in 100 mL of distilled water was prepared in a bottom flask and heated for 20 min at 70 °C with continuous mild stirring using a hot plate. Thirty milliliters of the plant extract were added dropwise to the iron solution and heated to 60 °C for 30 min. The dark brownish color indicated the formation of iron oxide NPs. The rest of the experiment was the same.

### 3.3. Characterization of Fe_3_O_4_ NPs

The fabricated Fe_3_O_4_ NPs were characterized using various analytical techniques to determine their size, shape, functional groups, crystallographic structure, and elemental analysis. The characterization techniques include UV-Vis spectroscopy, scanning electron microscopy (SEM), Fourier transform infrared (FTIR, spectrum 65, Mirpur, Azad Jamu and Kashmir), X-ray diffraction (XRD), and energy dispersive X-ray spectroscopy (EDX). The optical qualities of fabricated Fe_3_O_4_ NPs were investigated and approved by utilizing a double-beam UV-Vis spectrophotometer (Optizen, 3220) with a spectral range of 200–500 nm. The FTIR spectra were used to determine bioactive agents amenable to fabricated NPs and their stabilization in the range of 4000–500 cm^−1^. The XRD (Quaid-i-Azam University Islamabad, Pakistan) pattern of the synthesized NPs was revealed at 40 kV and 40 mA using CuKα radiation (1.5406) from 10–80° 2θ at 2°/step. The morphology and size of NPs were determined using the SEM technique (Institute of Space and Technology, Islamabad, Pakistan) operating at 10 kV. The EDX technique was used to determine the elemental composition of synthesized NPs, which was estimated by employing the EDX detector connected to the SEM instrument.

### 3.4. Measuring Antibacterial Activity

An antibacterial study of Fe_3_O_4_ NPs, algae, and plant extracts was performed using the agar well diffusion method against gram-positive (*B. pumilus* and *S. aureus*) and gram-negative (*E. coli* and *P. aeruginosa*) bacteria. A stock solution of 1 g/10 mL of each sample was used to assess the antibacterial and antioxidant activities. The antimicrobial activity was evaluated in a sterilized laminar flow. The sterilized nutrient agar medium was poured into Petri dishes. The medium was allowed to cool naturally, and the bacterial inoculum was spread gently with the help of cotton buds over the nutrient agar surface. The 6–8 mm wells were made using a sterilized cork borer in the agar medium. The antibiotic rifampicin and sterilized water were used as positive and negative controls, respectively. The algal and plant extracts (20 µL) were used in two wells of their respective plates. Iron oxide NPs were added to the wells at 20 µL, 30 µL, and 40 µL. The Petri plates were sealed cautiously with parafilm. These plates were incubated in an incubator for a day at 37 °C. After a given period, the widths of the growth inhibition zones were scaled in millimeters.

### 3.5. Antioxidant Potential

A 0.12 mg sample of DPPH was measured on a spring balance and mixed with 83 mL of methanol. The reagent bottle containing DPPH was covered with aluminum foil and kept in the dark for some time. A solution containing 1 mg of ascorbic acid in 100 mL of distilled water was used as a standard (control). Five samples of iron oxide NPs in triplets had concentrations of 10 µg/mL, 20 µg/mL, 30 µg/mL, 40 µg/mL, and 50 µg/mL. Methanol with varying concentrations including 690 µg/mL, 680 µg/mL, 670 µg/mL, 660 µg/mL, and 650 µg/mL was added to the 10 µg/mL, 20 µg/mL, 30 µg/mL, 40 µg/mL, and 50 µg/mL of iron oxide NP samples, respectively. Subsequently, 800 µg/mL of DPPH was added to each aliquot of iron oxide NPs and placed in the dark for 30 min. A major change in color from violet/purple to yellow was observed because of the scavenging action of samples. The absorbance was recorded at 517 nm via a spectrophotometer. The antioxidant potential of NPs was compared with that of ascorbic acid. The scavenging activities for iron oxide NPs and ascorbic acid were calculated using the following formula (Equation (1)):

Scavenging activity (%) = (A control − A sample)/A control × 100(1)

The same protocol was followed to determine the antioxidant potential of both the algal and plant extracts. After measuring scavenging activity, the IC_50_ values for all the desired samples were calculated.

## 4. Conclusions

The significance of herbal medicine well recognized in the medical sector because of their multitudinous benefits and limited complications for human health. The current research involves the fabrication of iron oxide NPs via green chemistry by exploiting the extracts of *Spirogyra hyaline*, a green alga, and *Ajuga bracteosa*, a medicinal plant. The proposed critically active biomolecules are flavonol glycosides, neo-clerodane, diterpenoids, phytoecdysones, ergosterol, iridoid glycosides, and many other polyphenols. The antioxidant action of the respective NPs, as assessed via the DPPH assay, showed that plant-based Fe_3_O_4_ NPs have a stronger ability to restrict oxidative stress than algal-based Fe_3_O_4_ NPs. Moreover, multidrug-resistant bacteria enhance the various infections that day-by-day lead to greater mortality around the world. The current biofabricated Fe_3_O_4_ NPs may provide an appealing alternative to combat bacteria or act as vehicles for the targeted delivery of drugs. In the current work, it is evinced that both algal- and plant-based Fe_3_O_4_ NPs exhibited remarkable antibacterial potency against both gram-positive and gram-negative bacteria. Plant-based Fe_3_O_4_ NPs showed many bactericidal effects that were fatal to gram-positive and gram-negative bacterial strains. The efficient antioxidant and remarkable antibacterial actions of plant-based NPs compared to algal-based NPs might be due to a higher number of various bioactive agents in the plant extract. The present study emphasized the importance of green-fabricated Fe_3_O_4_ NPs in the biomedical field, particularly as a potent antioxidant and an effective antimicrobial agent.

## Data Availability

We declared that the materials described in the manuscript, including all relevant raw data, will be freely available to any scientist wishing to use them for noncommercial purposes, without breaching participant confidentiality.

## Sample Availability

Samples of the compounds are available from the first authors.
